# The impact of municipal healthcare setting on the quality of end-of-life care: a population-based cohort study

**DOI:** 10.1186/s12904-026-02186-x

**Published:** 2026-06-10

**Authors:** Karin Dalhammar, Maria E. C. Schelin, Marie-Louise Möllerberg

**Affiliations:** 1 Faculty of Health and Society, Department of Care Science, Malmö University, Malmö, Sweden; 2 Institute for Palliative Care, Region Skåne and Lund University, Lund, Sweden; 3 Department of Clinical Sciences, Lund University, Lund, Sweden

**Keywords:** End-of-life care, Care setting, Community, Municipal healthcare services, Palliative care, Quality

## Abstract

**Background:**

In Sweden, approximately half of all individuals who die receive municipal care prior to death. Access to high-quality palliative care remains unequal, with known disparities between both care settings and diagnostic groups. Individuals receiving municipal home care are at higher risk of acute hospital admissions and in hospital death than those in long-term care facilities. However, the impact of municipal care setting on the quality of end-of-life care is largely unknown. The aim of this study was to describe the impact of care setting on the quality of end-of-life care among patients receiving municipal healthcare services.

**Method:**

This population-based cohort study included all individuals reported during 2023–2024 in the Swedish Register for Palliative Care as recipients municipal palliative care during the last week of life (*n* = 50,219). Associations between care setting (ordinary housing, and long- and short-term care facilities) and end-of-life quality indicators were analyzed using modified Poisson regression to estimate risk ratios (RR) with 95% confidence intervals. Analyses were adjusted for age, sex, and cause of death.

**Results:**

Compared to individuals receiving care in ordinary housing, those in long- and short-term care were more likely to receive symptom assessments ([RR; 95% CI] 1.14; 1.09–1.19 vs. 1.08; 1.03–1.13). However, they were less likely to receive end-of-life information ([RR; 95% CI] 0.92; 0.91–0.94 vs. 0.98; 0.97–0.99), bereavement support for relatives ([RR; 95% CI] 0.98; 0.97–0.99 vs. 0.95; 0.94–0.96), and external pain consultations ([RR; 95% CI] 0.52; 0.49–0.57 vs. 0.81; 0.76–0.88).

**Conclusions:**

Care setting influences the quality of municipal end-of-life care. Strengths and weaknesses vary across settings, highlighting the need for targeted quality improvement efforts.

## Background

The proportion of elderly people in the global population has been rapidly increasing, which poses challenges for end-of-life (EoL) care [1]. The expected demographic shift will increase the demand for municipal EoL healthcare, complicating efforts to ensure the quality of such care [2]. This challenge calls for integrative models of care that combine palliative, geriatric, and rehabilitative approaches, as recommended by Van den Block et al. (2025) in a European Delphi study across 28 countries [3].

In Sweden, about 94, 000 people die annually, and most of these deaths occur within municipal care settings, including ordinary housing, short-term facilities and long-term residential care facilities [4, 5]. As most people with palliative care needs receive care within the municipal care system during the final phase of life [6], municipal healthcare settings constitute the primary arena for EoL care. Palliative care aims to relieve suffering and improve quality of life for patients and families facing life-limiting illness [7]. Most individuals in municipal healthcare have basic palliative care needs, met through general palliative principles integrated into routine care, by regular staff [8]. For individuals with complex care needs, specialized palliative care may be provided by regional healthcare services, where multiprofessional teams, in collaboration with the municipality’s regular staff, offer comprehensive care and serve as an additional layer of support [9].

European recommendations for EoL care emphasize integrated, person-centered care [3]. Swedish policies align with these recommendations, stating that EoL care should be equitable and based on individual needs, irrespective of patients’ diagnosis, sociodemographic characteristics, and care setting [10]. Nevertheless, quality differences persist; older patients are less likely to receive EoL conversations and pain assessments than younger patients [11], and individuals with frailty and heart failure are less likely to receive palliative care consultations and EoL conversations and have a higher risk of dying in intensive care units compared to patients with cancer [12, 13]. While the quality of EoL care is higher in municipal healthcare services compared to hospitals, it remains inferior to that of specialized palliative care [14, 15].

In Sweden, municipal healthcare is organized as an integrated system supporting individuals with healthcare needs across different care settings. Nurses are responsible for nursing care and are supported by physicians from regional services, who retain overall responsibility for medical care and medical decision-making, while nursing assistant provide day-to day care, monitor patients´condition and report changes in needs or symptoms, including performing delegated tasks under the nurse supervision [16].

However, investigating the potential discrepancies in the quality of EoL care must account for the structural differences between care settings. Municipal healthcare includes nursing and medical care delivered either in institutional settings (long- or short-term facilities) or in private homes (here referred to as “ordinary housing”), depending on patients’ physical, functional, and social needs. Institutional care offers 24/7 support by on-site staff, whereas home care is provided through scheduled home visits and in response to acute care needs [17]. Consequently, institutional care can benefit from in-house resources, such as personnel and materials, whereas non-institutional care may rely on collaboration with informal caregivers and require greater adaptation to the home environment [18]. Barriers to providing palliative care vary between settings. For instance, home care predominantly faces organizational challenges, whereas care in nursing homes faces issues related to care content [19]. The organizational challenges healthcare personnel have reported in providing EoL home care include physical and professional distance from colleagues and limited access to multiprofessional expertise, which makes collaboration in complex situations particularly difficult [18, 20]. Moreover, research shows that individuals receiving EoL home care have a higher risk of acute hospital admissions and in-hospital mortality than those in long-term care [21–24]. However, they are more likely to receive EoL information and pain relief than residents in long-term care facilities [23, 25, 26]. This suggests that while some care dimension of EoL care may be more favorable in home care, other aspects of care quality may be compromised, underscoring the need to examine quality of EoL care across multiple dimensions and settings within the same healthcare system.

Despite municipal healthcare being the main arena for EoL care, quality differences across its settings remain largely unexplored. A broader understanding of potential contextual influences is essential for identifying structural inequities and informing targeted quality improvement efforts.

In this study, quality of EoL care was operationalized using a set of established process indicators reflecting core components of high-quality palliative care. These indicators include symptom assessment and management, Eol communication, bereavement support for next-of-kin, companionship at death, and avoidance of non-beneficial interventions in the last days of life. The selected indicators are derived from the Swedish Register of Palliative Care and are aligned with the Swedish National board of Health and Welfare´s national guidance for palliative care, which defines evidence-based quality indicators for EoL care [10]. Moreover, there is strong international agreement that these domains constitute key elements of EoL care quality, although their operationalization may vary across settings and contexts [27].

### Aim

This study aimed to describe the impact of care setting on the quality of end-of-life care among patients receiving municipal healthcare services.

## Methods

### Design

This population‑based cohort study used national quality register data to examine associations between care setting (long‑term care, short‑term care, and ordinary housing) and end‑of‑life quality indicators.

### Study population

**Fig. 1:**
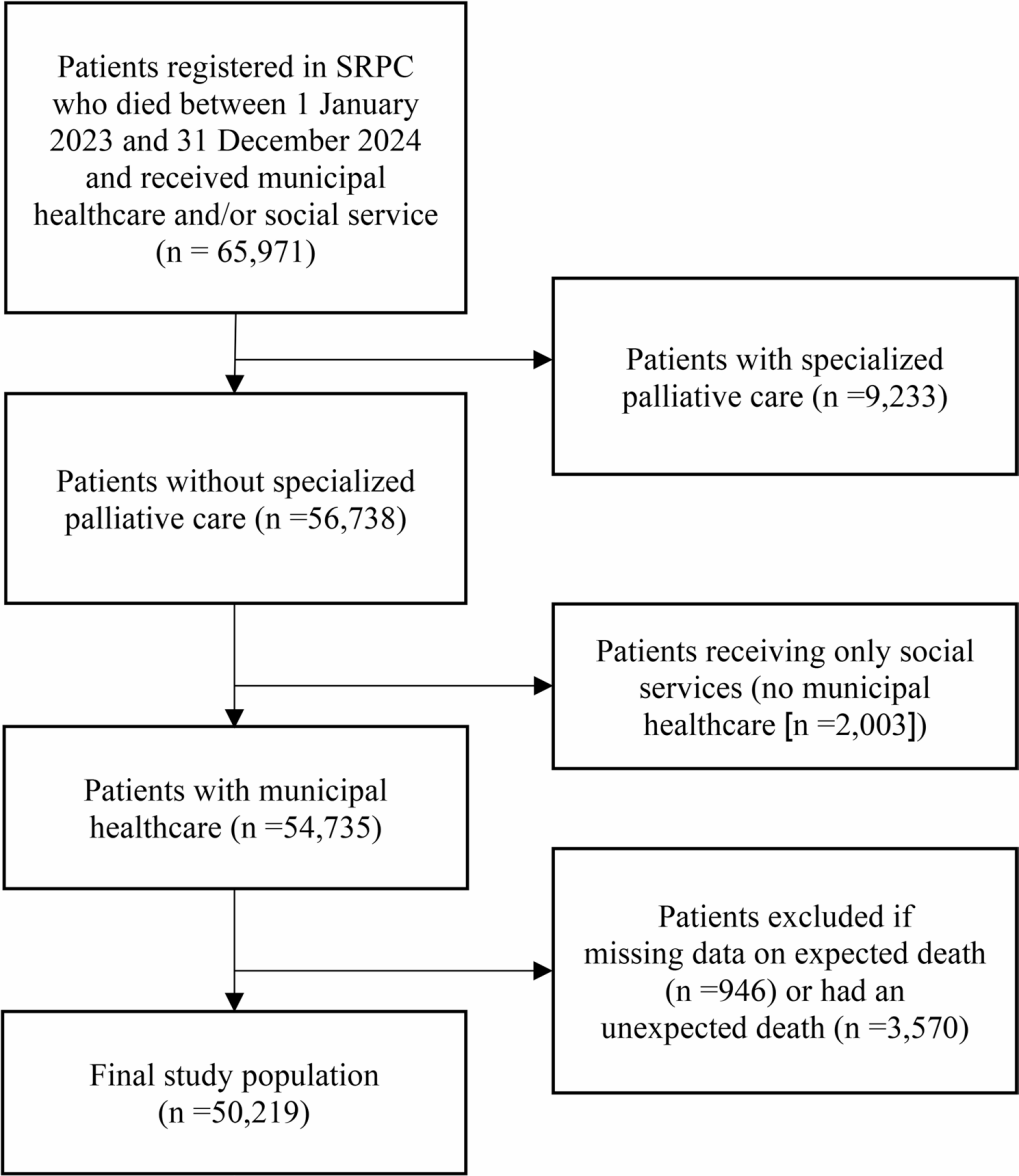
Flow chart of inclusion in the analysis

The study population comprised all adults registered in the Swedish Register of Palliative care (SRPC) between 1 January 2023 and 31 December 2024 who had an expected death and received municipal healthcare without support from a specialized palliative care service (*n* = 50,219) (Fig. 1). Patients receiving specialist palliative care were excluded to ensure a more homogeneous study population and to enable comparisons within municipal healthcare settings.

### Data collection

All data were obtained from the SRPC, a national quality register that includes information about the quality of EoL care during the final week of life. The register data are gathered through patient-level questionnaires completed by healthcare professionals after the patient’s death. The questionnaire items are based on the British Geriatric society’s principles for high-quality EoL care [28]. The completeness of SRPC is 71% for short-term care and nursing homes and 36% for ordinary housing [29]. The SRPC was linked to the Swedish Cause of Death Register at the time of data extraction. The Cause of Death Register is maintained by the Swedish National Board of Health and Welfare and includes mandatory information on all deaths in Sweden, including the underlying cause of death as classified in the International Classification of Diseases and Related Health Problems (ICD-10) [30]. From the linked dataset, we collected information on sex, age, underlying cause of death (according to ICD-10), and the setting where the EoL care was provided; ordinary housing (municipal healthcare service in private homes), short-term care facility **(**municipal healthcare service in a temporary residential care unit with around-the-clock on-site staff availability ), and long-term residential care facility (municipal healthcare service in a long-term residential care unit with around-the-clock on-site staff availability). We also obtained data on the quality of EoL care, including the assessment of oral health, pain, and other symptoms; the provision of EoL information by a physician; the provision of bereavement support for the next-of-kin; non-administration of parenteral fluid therapy; someone present at death (refers to the presence of any person at the time of death): and the provision of external consultation for symptom relief (refers to consultation by specialists outside the municipality’s regular staff, such as regional healthcare providers, to relieve symptoms and manage problems). These indicators are grounded in the national guidance and quality indicators defined by the Swedish National Board of Health and Welfare [10].

### Data analysis

The study population was categorized into three groups based on the municipal healthcare setting during the final week of life: ordinary housing, short-term care facility, and long-term care facility.

Descriptive and analytical statistics were used to analyze demographic and clinical data. Differences in means and proportions were analyzed using Anova for numerical data, and the chi-square test, and the Fisher exact test for nominal data.

The quality indicators regarding the provision of external consultation for symptom relief; non-administration of parenteral fluid therapy; someone present at death; and assessment of oral health, pain, and other symptoms were originally reported as “Yes”, “No”, and “Don’t know”. Responses marked “Don’t know” were excluded, and binary outcome variables were created. Similarly, the indicator on the provision of bereavement support was reported as “Yes”, “No”, “Don’t know” and “Had no known relatives”. A binary variable was created after the exclusion of “Don’t know” and “Had no known relatives” answers.

The indicator regarding EoL information provided by a physician was registered as “Yes”, “No”, “Don’t know”, “No, offered but declined”, “No, but by other healthcare personnel”, “No, lacks ability to participate”, and “EoL information offered to the next of kin”. The original response options were recoded into a binary variable for analysis. Responses indicating that the individual had received or been offered EoL information were coded as “Yes”, while responses indicating that the individual had not received such information were coded as “No”. End-of-life information delivered by non-physician healthcare personnel was coded as “No” as only information provided by a physician is classified as EoL information according to the Swedish National Bord of Health and Welfare [31]. The response “No, lacks ability to participate” was recoded based on the response “EoL information offered to the next of kin”; namely, if the latter was reported as “Yes”, the former was recoded as “Yes”; and if it was reported as “No”, the former was recoded as “No”. Responses marked as “Don´t know was excluded from the analysis (Table 1).

**Table 1:** Indicator recoded to binary variable reflecting if EoL information was provided by a physician

Binary outcome variable	The original response option
Yes	“Yes”
	“No, offered but declined”^a^
	“No, lacks ability to participate” in combination with “EoL information offered to the next of kin” = ”Yes”
No	“No”
	” No, but by other healthcare personnel”^b^
	“No, lacks ability to participate” in combination with “EoL information offered to the next of kin” = ”No”

^a^ The individual was considered to have been given the opportunity to receive EoL information. ^b^ The information was not given by the designated person responsible (physician)

To investigate the impact of municipal healthcare setting on each quality of EoL care outcome, we used modified Poisson regression to estimate the risk ratio (RR) with 95% confidence intervals (CIs) [32]. The RR indicates how much less (or more) likely an outcome is in one category compared to its reference category, which in this analysis was “Ordinary housing”. The statistical models were adjusted for the potential confounders, sex, age, and the underlying cause of death, as classified by the ICD-10. These adjustments were made because these factors are likely to affect both care quality and care setting. As data on main cause of death were missing for individuals who died in 2024, the adjusted analyses were restricted to cases with complete information and therefore based solely on individuals who died in 2023 (*n* = 26, 212).

A *p*-value of ≤ 0.05 was considered statistically significant. All statistical analyses were conducted using SPSS (version 30.0).

## Results

Of the 50,219 individuals included in this study, 29,667 (59.1%) were women (Table 1 presents detailed data along with percentages calculated per column). The mean age at the time of death was 86.3 years (SD ± 8.7). The predominant underlying causes of death were diseases of the circulatory system (28.4%), neoplasms (20.9%), and mental and behavioral disorders (15.1%). Regarding care setting, 35,378 (70.4%) individuals received care in a long-term residential care facility, 7,408 (14.8%) in a short-term care facility, and 7,433 (14.8%) in ordinary housing (Table 2).

**Table 2:** The characteristics of the study population and a comparison between municipal healthcare settings

	Total *n* = 50,219	Short-term care facility *n* = 7,408 (14.8%)	Long-term residential care facility *n* = 35,378 (70.4%)	Ordinary housing *n* = 7,433 (14.8%)	*p*-value
Age at time of death (mean ± sd)	86.3 ± 8.7	83.4 ± 9.1	87.4 ± 8.0	83.9 ± 10.6	< 0.001
Men, n (%)	20,552 (40.9)	3,753 (50.7)	13,200 (37.3)	3,599 (48.4)	< 0.001
Women, n (%)	29,667 (59.1)	3,655 (49.3)	22,178 (62.7)	3,834 (51.6)	
Main cause of death,^a^ n (%)	*n* = 26,212^b^				< 0.001
Neoplasms	5,483 (20.9)	1,853 (46.2)	2,028 (10.8)	1,602 (46.4)	
Endocrine, nutritional, and metabolic diseases	930 (3.5)	115 (2.9)	687 (3.7)	128 (3.7)	
Mental and behavioral disorders	3,947 (15.1)	192 (4.8)	3,635 (19.4)	120 (3.5)	
Diseases of the nervous system	3,620 (13.8)	176 (4.4)	3,304 (17.6)	140 (4.1)	
Diseases of the circulatory system	7,443 (28.4)	962 (24.0)	5,550 (29.6)	931 (27.0)	
Diseases of the respiratory system	1,215 (4.6)	189 (4.7)	846 (4.5)	180 (5.2)	
Diseases of the genitourinary system	507 (1.9)	95 (2.4)	335 (1.8)	77 (2.2)	
Symptoms, signs, and abnormal clinical and laboratory findings, not elsewhere classified	926 (3.5)	77 (1.9)	751 (4.0)	98 (2.8)	
Others	2141 (8.2)	350 (8.7)	1615 (8.6)	176 (5.1)	

^a^ According to ICD-10 classification

^b^ Information on the main cause of death is missing for individuals who died during 2024

### Quality of end-of-life care by municipal healthcare setting

Pain assessment was more likely to be reported in short-term care facilities (RR 1.06; 95% CI 1.02–1.09) and long-term residential care facilities (RR 1.07; 95% CI 1.04–1.10), compared to ordinary housing. Similarly, the assessment of other symptoms showed higher likelihood in short-term care (RR 1.08; 95% CI 1.03–1.13) and long-term care (RR 1.07; 95% CI 1.04–1.10). Oral health assessment was also more frequently reported in short-term care (RR 1.15; 95% CI 1.10–1.20) and long-term care (RR 1.16; 95% CI 1.12–1.20), (Table 3).

On the other hand, the provision of EoL information by a physician was less likely to be reported in short-term care (RR 0.98; 95% CI 0.97–0.99) and long-term care (RR 0.92; 95% CI 0.91–0.94) than ordinary housing. Bereavement support for the next-of-kin also showed lower likelihood in short-term care (RR 0.95; 95% CI 0.94–0.96) and long-term care (RR 0.98; 95% CI 0.97–0.99). Moreover, external consultation for symptom relief was less likely in short-term care (RR 0.81; 95% CI 0.76–0.88) and long-term care (RR 0.52; 95% CI 0.49–0.57). To have someone present at death was less likely in short-term care (RR 0.94; 95% CI 0.92–0.95) and long-term care (RR 0.95; 95% CI 0.94–0.96). Non-administration of parenteral fluid therapy was less likely in long-term care (RR 0.99; 95% CI 0.98–0.99) than ordinary housing (Table 3).

All comparisons were made using ordinary housing as the reference category. Full results, including unadjusted estimates and *p*-values, are presented in Table 3.

**Table 3:** The quality of end-of-life care by municipal healthcare setting

	RR (95%CI)	*p*-value	RR (95%CI)	*p*-value
	Unadjusted		Adjusted^a^	
EoL information provided by a physician				
Short-term care facility	0.99 (0.97–1.00)	0.006	**0.98 (0.97–0.99)**	**0.022**
Long-term residentia**l** care facility	0.89 (0.88–0.90)	**< 0.001**	**0.92 (0.91–0.94)**	**< 0.001**
Ordinary housing	1.00 (reference)		1.00 (reference)	
Pain assessment				
Short-term care facility	1.05 (1.03–1.07)	< 0.001	**1.06 (1.02–1.09)**	**< 0.001**
Long-term residentia**l** care facility	1.06 (1.04–1.08)	< 0.001	**1.07 (1.04–1.10)**	**< 0.001**
Ordinary housing	1.00 (reference)		1.00 (reference)	
Symptom assessment				
Short-term care facility	1.04 (1.01–1.07)	0.025	**1.08 (1.03–1.13)**	**0.002**
Long-term residentia**l** care facility	1.09 (1.07–1.12)	< 0.001	**1.14 (1.09–1.19)**	**< 0.001**
Ordinary housing	1.00 (reference)		1.00 (reference)	
Oral health assessment				
Short-term care facility	1.10 (1.07–1.13)	< 0.001	**1.15 (1.10–1.20)**	**< 0.001**
Long-term residentia**l** care facility	1.16 (1.13–1.18)	< 0.001	**1.16 (1.12–1.20)**	**< 0.001**
Ordinary housing	1.00 (reference)		1.00 (reference)	
External consultation for symptom relief				
Short-term care facility	0.81 (0.76–0.86)	< 0.001	**0.81 (0.76–0.88)**	**< 0.001**
Long-term residentia**l** care facility	0.30 (0.28–0.32)	< 0.001	**0.52 (0.49–0.57)**	**< 0.001**
Ordinary housing	1.00 (reference)		1.00 (reference)	
Non-administration of parenteral fluid therapy				
Short-term care facility	1.00 (0.99–1.00)	0.520	0.99 (0.98–1.00)	0.187
Long-term residentia**l** care facility	1.00 (0.99–1.00)	0.399	**0.99 (0.98–0.99)**	**< 0.001**
Ordinary housing	1.00 (reference)			
Someone present at death				
Short-term care facility	0.95 (0.93–0.96)	< 0.001	**0.94 (0.92–0.95)**	**< 0.001**
Long-term residentia**l** care facility	0.96 (0.96–0.97)	< 0.001	**0.95 (0.94–0.96)**	**< 0.001**
Ordinary housing	1.00 (reference)		1.00 (reference)	
Bereavement support offered to the next of kin				
Short-term care facility	0.94 (0.93–0.95)	< 0.001	**0.95 (0.94–0.96)**	**< 0.001**
Long-term residentia**l** care facility	0.98 (0.97–0.99)	< 0.001	**0.98 (0.97–0.99)**	**< 0.001**
Ordinary housing	1.00 (reference)		1.00 (reference)	

Bold values indicate statistically significant values (≤ 0.05)

^a^ Due to missing data on main cause of death (2024), analyses were restricted to individuals who died in 2023 (*n* = 26,212). Adjusted for; age, sex, and main cause of death, as classified in the ICD-10

## Discussion

This population-based study investigated the quality of EoL care among individuals receiving municipal healthcare in three different settings: ordinary housing and short- and long-term residential care facilities. Statistically significant differences were observed across all quality indicators, including clinical assessments, communication-related measures, and external consultations. However, none of the care settings appear to systematically provide superior quality EoL care relative to the others. These findings show that variation exists within the municipal healthcare system despite shared organizational structures.

While previous studies have primarily focused on variations in the quality of EoL care between different types of healthcare providers—such as hospital-based, municipality healthcare services, or Regionally provided specialized palliative care [14, 15]—this study introduces a novel perspective by examining differences between care settings operating under the same municipal healthcare provider. To our knowledge, this is the first study to approach the issue on an intra- organizational level.

This study found that individuals in short- and long-term residential care facilities were more likely to receive clinical assessments for oral health, pain and other symptoms, compared to those cared for in ordinary housing. This finding contrasts with a previous study showing that pain assessment instruments, such as verbal and numeric rating scales, are used less frequently in institutional care settings than ordinary housing [33]. Previous research has shown that higher academic qualifications and adherence to best practice among healthcare personnel are associated with improved symptom management, including more frequent symptom assessments [34, 35]. Therefore, the findings of the present study may be related to the competence and high educational level of municipality healthcare personnel working in institutional settings. In Swedish home care, nursing assistants carry a significant responsibility for providing EoL care on a day-to-day basis [36, 37]; however, the proportion of formally educated nursing assistants (licensed practical nurses) in ordinary housing is lower compared to long-term care facilities [38]. Accordingly, the lower level of formal education and perhaps limited knowledge of symptom assessment procedures may contribute to fewer assessments being performed in ordinary housing. This highlights the importance of building competence and clear routines for symptom assessment in homecare settings to ensure equitable and high quality EoL care.

Previous research on hospitalized patients shows that healthcare personnel conduct pain assessments primarily on account of patient’s self-reports of pain [39]. Therefore, the higher likelihood of symptom assessments in institutional settings may be attributed to the fact that healthcare personnel are more continuously present with the patient, which facilitates recognition of patient-reported symptoms or discomfort. In addition, healthcare personnel working in institutional settings perceive themselves as having more time for assessments compared to those working in ordinary housing [40]. In contrast, in ordinary housing, the presence of family members during the final stage of life may lead healthcare personnel to rely on their reports rather than validated tools [41]. This informal approach may reduce systematic assessments and contribute to an underdiagnosis of pain and oral health problems. Indeed, previous research reveals that home care recipients have poorer oral health than nursing home residents [42]. Qualitative research highlights that organizational factors, such as unclear responsibilities and limited training, hinder effective oral healthcare in both institutional and home settings [43, 44]. Taken together, these findings suggest that organizational and contextual factors, such as healthcare personnel presence, time availability, and established protocols, shape fundamental aspects of care, including symptoms and oral health assessments.

Given that pain assessment is the first step in effective pain management, research findings indicating that pain is more likely to be undertreated in long-term care than ordinary housing [26] appears somewhat inconsistent with our findings. Although healthcare personnel recognize the advantages of pain assessment [45], barriers to opioid administration persist due to insufficient training and misconceptions, such as concerns about patient addiction and adverse effects [46]. Perhaps these barriers to administration are more pronounced among healthcare personnel in long-term care.

Differences between settings are not limited to clinical assessments but extend to communication and support. Individuals in short- and long-term care facilities and their families were less likely to receive EoL information and bereavement support compared to those residing in ordinary housing. This finding is congruent with previous research [23, 25]. The greater probability of receiving adequate information on EoL care among individuals residing in ordinary housing may be related to the role of family involvement in EoL home care, which has been identified as a key factor in enabling older persons to remain in their own homes [47]. Family members often function both as a resource and as collaborative partners to formal care providers [48]. Consequently, healthcare personnel may feel a greater responsibility to communicate and provide bereavement support to ensure that the family members receive important information about the illness trajectory and support after the patient’s death. In addition, family involvement can drive the implementation of necessary measures, for instance, general practitioners report that input from relatives is an important trigger for initiating EoL conversations [49]. The lower likelihood of receiving EoL conversations, including information from a physician in short- and long-term care settings may be partly explained by the frequent daily interactions between the healthcare personnel and residents in these settings. Paradoxically, the frequency of interactions can make it harder to detect gradual health decline, according to previous research [50]. In contrast, the relatively less frequent contact with patients living in ordinary housing may make changes in health status more noticeable, thereby facilitating the timely initiation of EoL conversations. Another contributing factor may be time constraints. Previous research has shown that physicians frequently report lack of time as a barrier to initiating EoL conversations in primary care settings [51]. In residential care, physicians often operate under tightly scheduled visits, requiring them to prioritise their limited time across multiple patients, which may reduce opportunities for conducting EoL conversations. These differences in communication practices raise important questions about their potential impact on care outcomes. The provision of EoL information is intended to support decision-making and reduce unnecessary interventions, and their presence (or absence) may influence the pattern of healthcare utilization at the EoL.

Our study identified differences in key components of quality EoL care between settings, with individuals in ordinary housing being more likely to receive EoL conversation but less likely to receive symptom assessment compared with those in institutional care settings. Previous research has shown that both EoL conversation and systematic symptom assessment are associated with lower likelihood of acute care use in EoL [52, 53]. In addition, studies have reported higher levels of acute care use among individuals in ordinary housing compared with those in long-term care facilities [21–23]. Although healthcare utilization was not examined in the present study, our findings may help to contextualize these previous reported differences by highlighting how quality EoL care components linked to acute care use are differently distributed across municipal healthcare settings. Although acute care use at EoL is influenced by multiple factors [54, 55], previous research suggests that EoL conversations and symptom assessment are relevant components [52, 53]. Building on this evidence, as well as prior studies demonstrating differences in acute care use between care settings [21–23], a stronger focus on EoL conversations in long-term care facilities and greater attention to systematic symptom assessments in ordinary housing may be relevant when considering approaches to support appropriate use of healthcare services across settings.

Building on the communication aspect, this study also identified variations in the use of external palliative care consultations across settings. Compared to those living in ordinary housing, individuals in short- and long-term care facilities were less likely to receive external consultations. This finding aligns with prior research indicating that individuals receiving care in institutional settings, such as hospitals and long-term care facilities, have a lower likelihood of receiving palliative care consultation services compared to those living in ordinary housing [56]. One possible explanation for the lower use of palliative care consultations in short- and long-term care settings could be that healthcare personnel more frequently conduct symptom assessments, enabling early identification and management of problems before they become fully pronounced and require specialist consultation. For instance, research indicates that residents in long-term care facilities are more likely to receive palliative care support from their general practitioner than those in ordinary housing [24]. This could potentially reduce the need for external consultation or input from external specialists. However, previous research also shows that palliative care consultants can reduce potentially burdensome EoL transitions and acute care use among patients in long term-care settings [57]. This suggests that despite proactive symptom management, specialist palliative care input may still play an important role in improving EoL care quality and reducing hospitalizations at EoL.

Finally, the higher likelihood of patients dying in the presence of others in ordinary housing may attributed to family involvement in EoL care [18] and the known link between cohabitation and home death [58]. In contrast, individuals without close relatives may be more often referred to the hospital, suggesting that those who receive EoL care in ordinary housing form a selected group based on cohabitation status. Further research is needed on the role of family caregivers in the quality of EoL care, as our study lacks data on informal caregiving.

### Strengths and limitations

This study has several methodological strengths. First, the large, population-based sample (*n* = 50,219) enhances the generalizability of the findings within municipal home healthcare services in Sweden. Second, the use of data from the SRPC enabled the systematic analysis of multiple quality indicators across care settings. Third, the analyses were adjusted for key demographic and clinical variables, including age, sex, and main cause of death, strengthening the internal validity of the results.

However, certain limitations should be considered when interpreting the findings. As this is an observational study, unmeasured confounding cannot be completely excluded. Missing data on main cause of death in 2024 may have influenced the estimates if causes of death differed systematically from previous years. The coverage of the SRPC varies between settings, with lower completeness in ordinary housing (36%) compared to institutional care (71%). This may introduce selection bias if differences in the likelihood of registration between settings are related to differences in the quality EoL care, such bias, if present, could lead to associations being observed in the data material without being present in reality. Moreover, as the data are based on postmortem reporting by healthcare professionals, reporting may be influenced by retrospective assessment, introducing a risk of reporting bias related to recall and variability in documentation quality. Furthermore, the register lacks information on the qualifications of the healthcare personnel, staffing levels, and access to multidisciplinary support, all of which may influence care quality, but could not be adjusted for in the analysis. Finally, as this study focused on municipal care settings, this limits generalizability of the findings to patients receiving specialized palliative care.

## Conclusions

This study demonstrates that the quality of EoL care within municipal healthcare services varies depending on the care setting, despite being delivered under the same provider structure and without support from specialized palliative care. Recognizing these variations is essential for planning and delivering equitable EoL care. Differences across key quality indicators suggest that structural and contextual factors shape care practices in ordinary housing and in short- and long-term residential care facilities. Quality improvement efforts may benefit from being tailored to the specific setting. In short- and long-term care facilities, initiatives aimed at strengthening EoL communication, bereavement support, and access to external consultants for symptom relief could be relevant, whereas in ordinary housing, greater emphasis on systematic symptom assessment may be appropriate. Such targeted improvements may support the provision of quality EoL care, regardless of municipal healthcare setting. Future research should explore how personal resources, professional competence, and intervention timing influence care quality and outcomes across settings. In addition, studies examining the integration of specialist palliative care support into municipal care and its impact on hospital admissions and patient experience could guide future models of care.

## Data Availability

The datasets generated and/or analyzed during the current study are not publicity available due to regulations in the Swedish Ethical Act (2003:460) and the Swedish Data Protection Act (2018:218; 2019:219) but are available from the corresponding author on reasonable request.

## Abbreviations

EoL: End-of-life
CDR: Cause of Death Register
CI: Confidence Interval
ICD-10: International Classification of Diseases, 10th revision
RR: relative risk
SRPC: Swedish Register of Palliative care
